# Biomarkers of a five-domain translational substrate for schizophrenia and schizoaffective psychosis

**DOI:** 10.1186/s40364-015-0028-1

**Published:** 2015-02-06

**Authors:** Stephanie Fryar-Williams, Jörg E Strobel

**Affiliations:** The University of Adelaide, Adelaide, SA Australia; The Queen Elizabeth Hospital, Woodville, SA Australia; Basil Hetzel Institute for Translational Health Research, Woodville, SA Australia; Youth in Mind Research Institute, Norwood, SA Australia

**Keywords:** Biomarkers, Mental illness, Translational, Schizophrenia, Psychosis, Sensory processing, Noradrenaline

## Abstract

**Background:**

The Mental Health Biomarker Project (2010–2014) selected commercial biochemistry markers related to monoamine synthesis and metabolism and measures of visual and auditory processing performance. Within a case–control discovery design with exclusion criteria designed to produce a highly characterised sample, results from 67 independently DSM IV-R-diagnosed cases of schizophrenia and schizoaffective disorder were compared with those from 67 control participants selected from a local hospital, clinic and community catchment area. Participants underwent protocol-based diagnostic-checking, functional-rating, biological sample-collection for thirty candidate markers and sensory-processing assessment.

**Results:**

Fifteen biomarkers were identified on ROC analysis. Using these biomarkers, odds ratios, adjusted for a case–control design, indicated that schizophrenia and schizoaffective disorder were highly associated with dichotic listening disorder, delayed visual processing, low visual span, delayed auditory speed of processing, low reverse digit span as a measure of auditory working memory and elevated levels of catecholamines. Other nutritional and biochemical biomarkers were identified as elevated hydroxyl pyrroline-2-one as a marker of oxidative stress, vitamin D, B6 and folate deficits with elevation of serum B12 and free serum copper to zinc ratio.

When individual biomarkers were ranked by odds ratio and correlated with clinical severity, five functional domains of visual processing, auditory processing, oxidative stress, catecholamines and nutritional-biochemical variables were formed. When the strengths of their inter-domain relationships were predicted by Lowess (non-parametric) regression, predominant bidirectional relationships were found between visual processing and catecholamine domains. At a cellular level, the nutritional-biochemical domain exerted a pervasive influence on the auditory domain as well as on all other domains.

**Conclusions:**

The findings of this biomarker research point towards a much-required advance in Psychiatry: quantification of some theoretically-understandable, translationally-informative, treatment-relevant underpinnings of serious mental illness. This evidence reveals schizophrenia and schizoaffective disorder in a somewhat different manner, as a conglomerate of several disorders many of which are not currently being assessed-for or treated in clinical settings. Currently available remediation techniques for these underlying conditions have potential to reduce treatment-resistance, relapse-prevention, cost burden and social stigma in these conditions. If replicated and validated in prospective trials, such findings will improve progress-monitoring and treatment-response for schizophrenia and schizoaffective disorder.

## Background

The architecture of schizophrenia is still largely unknown and conventional systems for diagnosing schizophrenia and schizo-affective states are still based on phenomenological descriptions of symptoms and behaviours [1]. While progress has been made regarding the underlying molecular biology and neuropathology of schizophrenia, its characterization has not improved beyond coarse division of its symptoms into “positive” and “negative” types that may not truly reflect underlying neurobiological mechanisms [2]. Finding biomarkers, directly linked to the underlying structure of schizophrenia, could be a great leap forward for Psychiatry so the search for peripheral and central markers for these serious mental illness conditions and for affective disorders has been underway for many years [3]. Many uni-dimensional studies have tested specific biomarkers based on the hypotheses of monoamine, neuro-immune-inflammatory, neuroendocrine and neuroplasticity dysfunction [4]. Other studies have tested the ability of multi-analyte technology to profile biomarkers. These techniques have the capacity to generate thousands of markers that may demonstrate overall predictive power but yield only marginal understanding of the disease process and no causal framework for practical application to assessment and treatment in the clinical context [5].

Against this background of heterogeneity, overlapping co-morbidity and fading faith in the validity of descriptive classification systems [6], schizophrenia and related psychotic conditions are increasingly viewed as complex, polygenic diseases involving hundreds of forms of functional pathology. For this reason, investigation of putative biomarkers across a number of functional domains, may better reflect heterogeneity and assist to identify sub-phenotypic areas within the schizophrenia condition. Moreover, in the clinical setting, insufficient presenting symptoms that fail to meet criteria for any firm diagnosis, cause primary-care clinicians considerable stress. They must walk a fine line between knowing that the earlier schizophrenia is diagnosed and treatment begun, the better the outcome may be [7] and knowing that un-necessary cost and distress may accompany an inaccurate diagnosis [8]. For both reasons quantitative confirmation of the underlying causal components of serious mental illness is urgently required, in order to augment professional judgement about accuracy of diagnosis and clinical management of the presenting condition [9].

**Figure 1:**
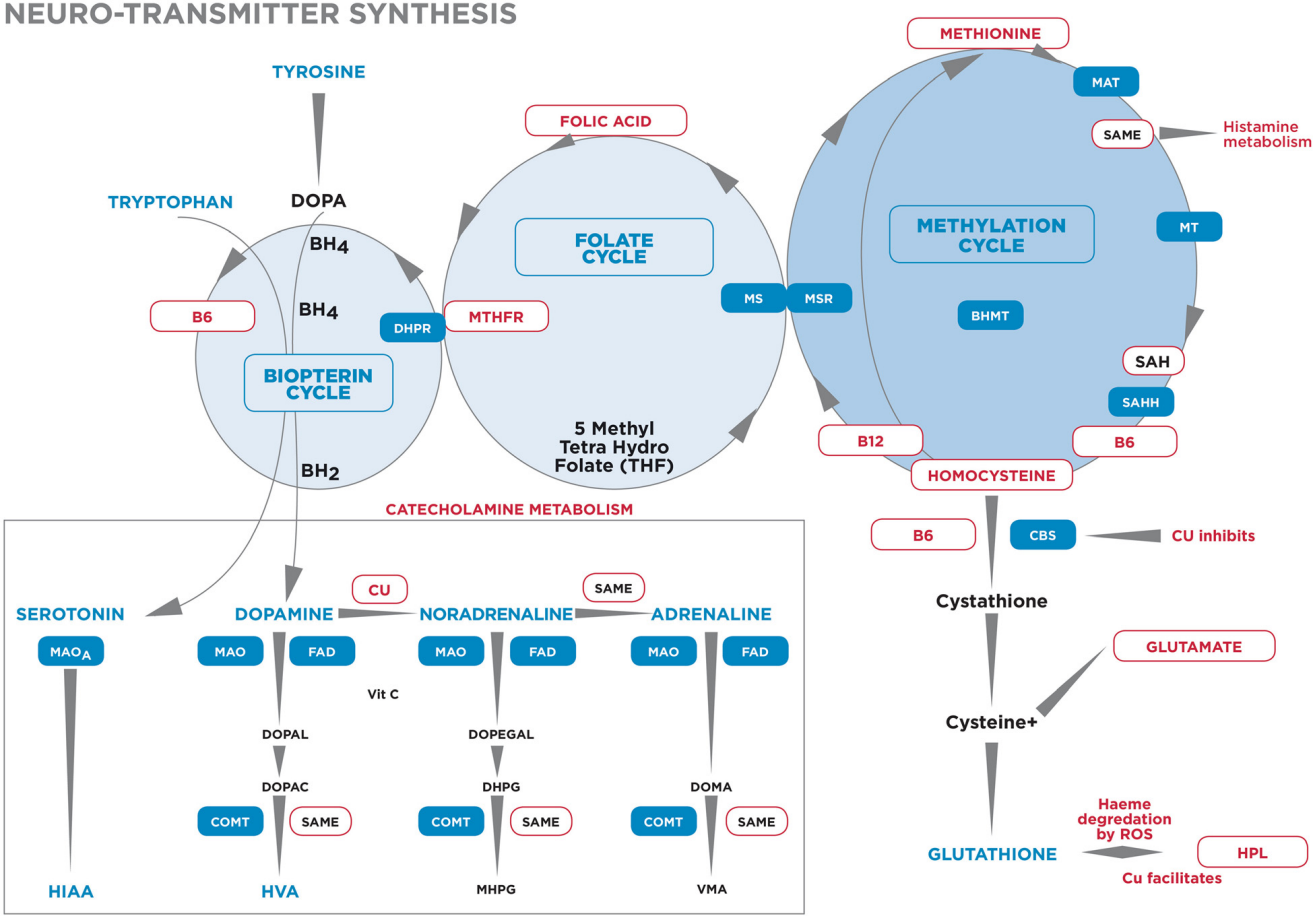
**Cofactor vitamins and minerals in relationship to catecholamine biochemistry.** **DOPA** – dihydroxyphenylalanine, **BH4** - tetrahydrobiopterin **BH2** – dihydrobiopterin, **MTHFR**- Methylenetetrahydrofolate reductase, **MAT** - Methionine adenosyltransferase, **SAMe** - S-Adenosylmethionine, **MT** - Methyltransferase, **SAH**- S-Adenosylhomocysteine, **SAHH** - S-Adenosylhomocysteine-hydrolase, **CBS** - Cystathione Beta Synthetase, **BHMT** - Betainehomocysteine methyltrasferase, **DMG** - Dimethylglycine, **TMG** - Trimethylglycine, **MSR** - Methionine sulphoxide reductase, **MS** - Methionine synthase, **5HIAA** – 5-hydroxyondolacetic acid, **HVA** – homovanillic acid, **MAO**- monoamineoxidase., **MHMA** - 3-methoxy-4-hydroxymandelic acid = **VMA**-Vanillylmandelic acid, **FAD** - flavin adenine dinucleotide, **MHPG** -4-hydroxy-3-methoxyphenylglycol, **DOPAL** – dihydroxyphenylacetaldehyde, **DOPAC** - dihydroxyphenylacetic acid, **DOPEGAL** – dihydroxyphenylglycolaldehyde, **DOMA** – dihydroxymandelic acid **DHPG** – dihydroxyphenylglycal, **COMT**- catechol-o-methyl-transferase.

Schizophrenia and schizoaffective disorder are allied conditions within the clinical setting. DSM IV-TR classification system describes schizoaffective disorder as a major mood disorder episode occurring concurrent with symptoms that meet characteristic symptoms for schizophrenia [10]. The Mental Health Biomarker Project (2010–2014) sought to investigate candidate markers with a high possibility of being associated with schizophrenia or schizo-affective disorder aetiology, across a number of putative functional domains. These domains included neurotransmitter synthesis and metabolism, oxidative stress, nutrition (vitamin and mineral enzyme co-factors), visual and auditory information processing. This project’s inquiry in the biochemical domain has a theoretical basis explained by remote and proximal pathways related to monoamine synthesis and metabolism and an overview of this biochemistry landscape is depicted in Figure 1. In undertaking this research, we considered it important to use accessible, commercially-available laboratory tests, easily-procurable inexpensive equipment and simple-to-use assessment methods that could be conducted in a 45 minute low ambient noise clinic consultation.

## Results

### Patient characteristics

In this retrospective case–control study, 67, DSM-IV-R diagnosed [11] participants, between 18 and 60 years of age were enrolled from the western catchment area of Adelaide, South Australia. In order to exclude as many confounding variables as possible, participants were subject to multiple selection exclusion criteria as outlined in the Methods section. A small data set was therefore obtained and data analysis was carried out on hundred and thirty four participants (67 cases and 67 controls). The sample was analysed as total cases versus total controls and no separate analysis by age and sex was conducted due to sample size. Baseline case–control sample characteristics for age and sex, BMI, urine creatinine, near vision, auditory thresholds and in Table 1.

**Table 1:** **Baseline characteristics of case and control groups (n 67)**

**Parameter**	**Number patients**	**Mean patients**	**Standard error**	**Number controls**	**Mean controls**	**Standard error**
Age participants	67	40.5	1.3	67	45.7	1.4
Age males	37	38.6	1.4	33	46.4	1.7
Age females	30	42.9	2.4	34	45.0	2.2
Body mass index (BMI)	53	30.0	1.2	66	26.7	0.6
Right hand dominance %	65	92.6	2.1	67	93.1	1.7
Urine creatinine (mmol/L)	66	9.2	0.7	67	9.5	0.7
% hydroxyl indole acetic acid (5-HIAA)	66	4.3	0.8	67	1.6	0.1
Plasma homocysteine (umol/L)	66	10.0	0.3	66	9.5	0.3
Red cell acetylcholine esterase (U/gb Hb)	61	39.8	0.7	67	39.6	0.7
Hearing threshold (Db)	60	533.3	33.3	64	515.6	24.8
Visual threshold of near vision	61	6.3	0.5	67	5.2	0.1

### Candidate marker results

Figure 1 depicts the relationship between candidate markers for which results are given. These candidate markers exist in biochemical pathways which have potential to exert subtle, yet cumulative effects on catecholamine neurotransmitter synthesis and metabolism via deficits or excesses in enzyme cofactors and intermediate substances related to folate and methionine (one-carbon) cycles [12-14]. Results related to catecholamines were for urine dopamine, noradrenaline, adrenaline, homovanillic acid (HVA), methoxy-hydroxymandelic acid (MHMA), 5-hydroxyindoleacetic acid (5-HIAA) [15], creatinine and hydroxyhemopyrroline-2-one (HPL) - a metabolite indicative of oxidative stress [16]. Results related to vitamins and mineral enzyme cofactors and intermediate substances were for vitamin D [17], these vitamin B6, B12 [18,19], folate [20], copper and red cell zinc [21,22], homocysteine [23], ceruloplasmin [24], histamine [25], and methyltetrahydrofolate reductase (MTHFR 677 C- > T) gene polymorphism [26]. Participants were also given a number of auditory and visual assessments for distance vision, visual span, competing words (dichotic listening), reverse digit span (working memory) and visual and auditory speed of processing (as a percent of age).

### ROC analysis

Receiver Operating Curve (ROC) variables [27,28], were analysed using XLSTAT software [29], however many of these variables lacked normal distribution and their characteristics, as determined by EasyFit software [30] are outlined in Table 2.

**Table 2:** **Variables distribution summary for biomarkers**

**ROC variable**	**No Obs**	**Obs. without missing data**	**Min-imum**	**Max-imum**	**Mean**	**Standard ** **deviation**	**Median**	**Mean absolute deviation**	**Fitted distribution**
**Visual domain**									
Visual span	134	126	0.00	8.00	5.476	1.35	6.00	1.00	Pert
Visual speed of processing discrepancy (% of age)	134	122	−90.00	207.69	6.01	54.44	−5.55	30.24	Log Normal
Distance vision on right	134	128	0.00	36.00	7.98	6.03	6.00	1.50	Chi-square
**Auditory domain**									
Reverse digit span	134	127	2.00	8.00	4.22	1.33	4.00	1.00	Gamma (3P)
Competing words discrepancy (% of pass score)	134	124	−69.23	50.00	0.55	22.61	3.84	15.38	Johnson SB
Auditory speed of processing discrepancy (% of age)	134	121	−100.00	220.00	−3.83	56.68	−18.00	31.12	Gen. Extreme Value
**Catecholamine domain**									
Dopamine	134	133	45.00	358.00	142.47	53.64	129.0	32.00	Log-Logistic
Noradrenaline	134	133	3.00	106.00	25.278	18.53	19.00	9.00	Johnson SB
Adrenaline	134	133	0.00	27.00	4.413	5.10	2.00	1.00	Log-Logistic
**HPL/Creatinine model**	134	133	0.35	40.04	4.586	5.94	2.47	1.18	Burr
**Nutrition-Biochemistry domain**									
Free copper to Zinc ratio	134	133	−1.85	1.60	0.267	0.52	0.31	0.31	Log-Logistic
B6 activation	134	129	12.80	1570.0	140.44	164.6	90.00	25.00	Gen. Extreme Value
Red cell folate	134	133	506.00	3291.0	1788.9	448.7	1733.	236.00	Log-Logistic
Serum B12	134	134	42.00	1388.0	406.15	178.5	367.0	104.00	Gen. Extreme Value
Vitamin D	134	132	13.00	149.00	52.462	22.15	52.00	14.00	Dagum

Variables that were not successful as biomarkers on ROC analysis were: methyltetrahydrofolate reductase (MTHFR C667T) polymorphism, plasma homocysteine and urine metabolites homovanillic acid (HVA) and methoxy-hydroxymandelic acid (MHMA). Despite this, the homozygous MTHFR (677 T) polymorphism demonstrated a significant positive correlation with elevated homocysteine (n 132, rho 0.223, P 0.010 for levels > 12 umol/L). The serotonin metabolite, 5-hydroxyindoleacetic acid (HIAA), also failed to produce a sufficient area under the curve (AUC > 0.6). High histamine produced a poor quality ROC curve, however together with noradrenaline, produced a significant ROC AUC of 0.86. In the field of sensory-processing assessment, all putative variables tested reached ROC-identified biomarker status, with the exception of outcome measures for gap-detection, auditory-figure ground tests for auditory processing disorder and near vision on Sussex near vision test.

Fifteen main variables emerged from ROC analysis with an AUC of > 0.6 at the 95 percent level of significance and one variable (for vitamin B12) with a ROC of only 0.565 contributed to the overall strength of the biochemistry components. These variables were segregated into five functional domains (Table 3). The first domain contains measures of visual cognitive performance and processing (Visual domain). The second domain contains of measures of auditory cognitive performance and processing (Auditory domain). The third domain contains measures of catecholamines with potential for neurotransmitter action (Catecholamine domain). The fourth domain consists of the HPL/Creatinine level which is taken as a measure of haeme-related oxidative stress. The fifth domain consists vitamin and mineral cofactors for catecholamine synthesis and metabolism (Nutritional-biochemical domain).

**Table 3:** **ROC results for schizophrenia and schizoaffective disorder, with odds ratios**

**ROC variables and domains**	**No.Obs**	**AUC**	**Odds ratio**	**Odds ratio P**	**ROC variables and domains**	**No. Obs**	**AUC**	**Odds ratio**	**Odds ratio P**
Low visual span	126	0.862	22.46	< 0.0001	High Dopamine	133	0.702	9.60	<0.0001
High visual speed of processing discrepancy (% of age)	122	0.875	27.22	< 0.0001	High Noradrenaline	133	0.851	21.25	< 0.0001
Poor distance vision on right	128	0.597	5.17	0.0001	High Adrenaline	133	0.844	14.32	< 0.0001
**Visual domain**	**120**	**0.915**	**41.48**	**< 0.0001**	**Catecholamine domain**	**133**	**0.859**	**4.12**	**<0.0001**
Reverse digit span	126	0.862	11.00	<0.0001					
High competing words discrepancy (% of pass score)	124	0.875	10.69	<0.0001	Low vitamin B6 activated	129	0.638	3.75	0.0009
High auditory speed of processing discrepancy (% for age)	121	0.874	21.23	<0.0001	High free copper to zinc ratio	133	0.611	2.60	0.0104
**Auditory domain**	**119**	**0.891**	**29.57**	**<0.0001**	Low red cell folate	133	0.654	3.64	0.0005
					Low vitamin D	132	0.651	3.24	0.0026
High (HPL/Creatinine) model	133	0.696	4.12	< 0.0001	High serum B12 (80% CI)	134	0.565	1.89	0.0933
**Oxidative stress domain**	**133**	**0.696**	**4.12**	**<0.0001**	**Nutrition-Biochemistry**	**126**	**0.797**	**8.5**	**< 0.0001**

Biomarker variables within the first, visual domain are visual (symbol) span, threshold visual speed of processing performance as a percentage of age and reduced distance-vision score on right (representing asymmetric binocular distance-vision acuity). When pooled together these biomarkers demonstrated strength on ROC analysis with AUC (0.915, P < 0.0001) with domain sensitivity of 89% and specificity of 85%.

Biomarker variables in the second, auditory domain are reverse digit span (measures verbal, auditory working memory), competing-words performance for age as a percentage of age (representing intra-cerebral dichotic processing of auditory information) and threshold speed of auditory processing as a percentage of age. When pooled together these biomarkers demonstrated ROC AUC (0.891, P < 0.0001) with domain sensitivity of 87% and specificity of 82%.

Biomarker variables within the third, catecholamine domain are in order of strength: Noradrenaline, Adrenaline and Dopamine. When pooled together these biomarkers demonstrated ROC analysis AUC (0.859, P < 0.0001) with domain sensitivity of 84% and specificity of 75%.

Within the fourth domain, hydroxyhaemopyrroline-2-one, the sole biomarker in the HPL/creatinine domain is identified as a marker of oxidative stress. It demonstrated an AUC of 0.696 with sensitivity of 70% and specificity of 64%.

Variables within the fifth, Nutrition domain are high serum B12 and ratio of free copper to red cell zinc. Also low red cell folate, activated B6 (Pyridoxal-5’-phosphate coenzyme form) and serum vitamin D (25-OH). When pooled together these biomarkers demonstrated ROC analysis AUC (0.797, P < 0.0001) with domain sensitivity of 55% and specificity of 88%.

### Odds ratio of diagnostic association and Spearman’s correlation analysis

Odds Ratio analysis may be used with ROC analysis in a case–control study to approximate the relative risk of particular pathological outcome measures being associated with a low period prevalence disorder such as schizophrenia or schizoaffective disorder [31] (Table 3). Despite this, some statisticians claim that odds of association results may be inflated by up to three times with a case–control design. [32]. Despite this empirical estimate, given that most ROC variables identified in our study achieved a robust odds ratio of much over 2, all domain odds ratios were checked for their capacity to survive a “division by three” test and those that passed this test fell into five domains. These are ranked below, in descending order:

Using Spearman’s correlation analysis, discrete symptom intensity ratings from the Brief Psychiatric Rating Scale (BPRS) [11] and the Positive and Negative Syndrome Scale for Schizophrenia (PANSS) [33], were summated for each participant’s clinical or subclinical symptoms, to give an overall Symptom Intensity Rating index (SIR). This index was taken as measure of clinical severity and investigated by Spearman’ correlates with respect to other ROC variables. On Spearman’s correlation analysis, all domains of interest correlated significantly with SIR at the 95 per cent level of significance and high correlations with the clinical severity (SIR) also fell into five ranked domains:

## Ranking strength of domains by Odds ratio (divided by three) and SIR-Spearman’s correlation

### Rank I: Visual biomarkers

Odds ratio: high visual processing speed % discrepancy for age (n 122, OR 9.07, P <0.0001), low visual span (n 126, OR 7.46, P < 0.0001).

SIR-correlates: low visual span (n 126, rho 0.605, P < 0.0001), delayed visual speed of processing (n 122, rho 0.575, P <0.0001).

### Rank II: Auditory biomarkers

Odds ratio: high competing words (dichotic listening) discrepancy (n 124, OR 10.67, P < 0.0001), high auditory speed of processing as a % of age (n 121, OR 7.08, P < 0.0001), (low reverse digit span (n 127, OR 3.7, P < 0.0001).

SIR correlates: delayed auditory speed of processing (n 120, rho 0.570, P < 0.0001), low competing words (dichotic listening) performance (n 124, rho 0.505, P <0.0001), (low reverse-digit span (n 128, rho 0.384, P <0.0001).

### Rank III: Elevated catecholamine biomarker domain

Odds ratio: elevated Noradrenaline (n 133, OR 7.08, P < 0.0001), elevated Adrenaline (n 133, OR 4.77, P < 0.0001), elevated dopamine (n 133, OR 3.2, P <0.0001).

SIR correlates: high noradrenalin (n 133, rho 0.575, P < 0.0001), high adrenaline (n 133, rho 0.455, P <0.0001), high histamine and NA (n 126, rho 0.427, P < 0.0001).

### Rank IV: HPL/Creatinine, oxidative stress domain

A significant correlative relationship also existed between the oxidative stress marker, elevated HPL/creatinine and SIR (n 133, rho 0.327, P < 0.0001), with correlations also found between elevated catecholamine levels and auditory and visual domain deficits (Table 4).

### Rank V: Overall nutrition-biochemistry domain

Odds ratio: n 126, OR 2.83, P < 0.0001.

SIR correlates: (n 126, rho, 0.404, P <0.0001).

SIR was also found to correlate with specific ROC variables for high noradrenaline (n 124, rho 0.381, P <0.0001, high adrenalin (n 124 rho 0.302, P 0.000) and high dopamine (n124, rho 0.301, P 0.000).

### Translational cross-domain relationships

On Spearman’s correlation analysis, all domains of interest inter- correlated significantly at the 95 per cent level of significance and inter-domain results are presented in Table 4.

**Table 4:** **Domain inter-correlations**

**Rho). ROC model (108 0bs)**	**Visual domain**	**Auditory domain**	**Catecholamine domain**	**HPL-Creatinine doman**	**Nutrition domain**
Visual domain n 120	1	**0.555**	**0.448**	**0.310**	**0.294**
Auditory domain n119	**0.555**	1	**0.481**	**0.337**	**0.384**
Catecholamine domain n 133	**0.448**	**0.481**	1	**0.298**	**0.319**
HPL/Creatinine n133	**0.310**	**0.337**	**0.298**	**1**	0.108
Nutrition domain n 126	**0.294**	**0.384**	**0.319**	0.108	1

Values in bold are different from 0 with a significance level of alpha = 0.05 and rho > 0.3 have a P value of < 0.0001.

**Figure 2:**
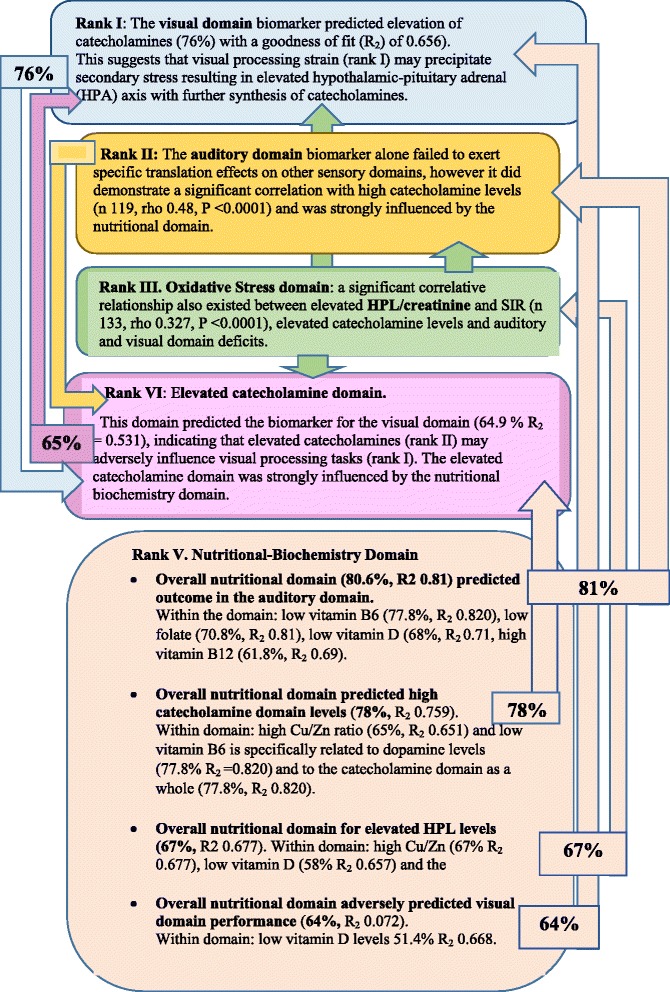
**Percentage translational relationships of strength-ranked biomarkers for schizophrenia and schizoaffective disorder, where R**_**2**_ **= goodness of fit.**

**Figure 3:**
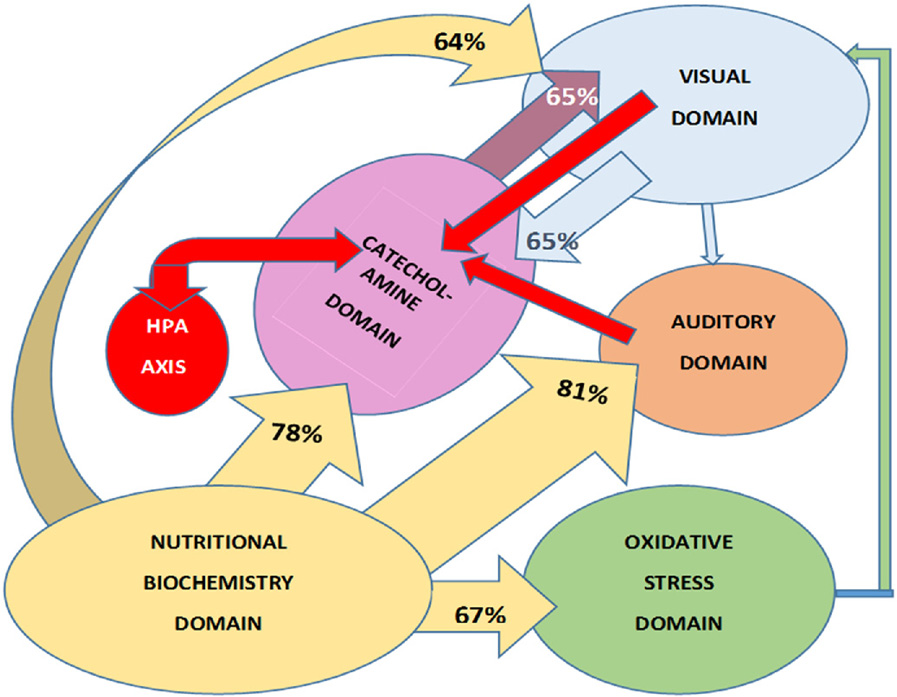
**Translational cross-talk between biomarker domains.**

Ranking of biomarker strengths into five ranked domains according to odds ratio and SIR-correlates, revealed interesting strength-related relationships between biomarker domains. However when ROC variable translational strengths were predicted by Lowess (non-parametric, locally-weighted) regression analysis [34] for which the ideal predictive probability is 60% or more, at goodness of fit (R^2^) of 0.6 or better. When results were presented within the framework of the previous five ranked domains, a similar - yet different view of ROC translational relationships emerged (Figures 2 and 3).

## Discussion

Obtaining these biomarkers of schizophrenia and schizoaffective disorder utilises readily-procurable laboratory tests, inexpensive equipment, and easy-to-use assessment methods that be conducted in a 45 minute consultation, by a health practitioner in a low ambient noise, everyday clinical setting. Outcome measures obtained are then ROC- analysed to define biomarkers that are then allocated to one of the five domains of neuro-biological inquiry. Together these represent aspects of schizophrenia and schizoaffective disorder that reveal new aspects of these conditions.

Moreover, odds ratio of diagnostic association and Spearman’s correlation with symptom severity (SIR) indicate that domains of function in schizophrenia and schizoaffective disorder rank into five descending strengths. Translational predictions from Lowess regression analysis preserve this ranking but reveal, (as would be expected), that the lowest-ranking nutritional-biochemical cellular domain exerts a powerful translational influence on all other biochemical and neurone-dependent sensory processing domains.

### Rank I: Visual domain

Reduced visual (symbol) span (a measure of visual attention and working memory), aged speed of visual processing, and deficit in distance vision on the right, were found to achieve biomarker status. When pooled together these biomarkers demonstrated the greatest strength on ROC analysis. On translational analysis, processing in the visual domain was found to be influenced by oxidative stress (HPL) and low levels of vitamin D, along with dysfunctions in overall nutrition-related biochemistry. Such prominent visual findings in schizophrenia and schizoaffective disorder highlight the need for more detailed visual assessment and correction of visual deficits for first episode psychosis patients, particularly since visual impairments have already been reported in psychosis [35], in both medicated and medication-free schizophrenic subjects [36,37]. With respect to the deficit findings related to the right distance vision in schizophrenia, it is known that right parietal cortex and right frontal cortex exert a stronger driving influence on visual areas of the left hemisphere [38].

The role of vitamin D in visual deficit is understandable in terms of its role in promoting glutamate decarboxylase (GAD 67) expression and therefore GABA production [39]. It also has a role in Glutathione production, with low levels of glutathione found in vitamin D deficient animals [40]. A reciprocal mechanism also exists between elevated catecholamines and low vitamin D levels, with catecholamines priming parathyroid hormone, to release calcium (Ca ++) ions from neuronal cells - an effect that is directly opposed by the action of vitamin D, theoretically leading to increased vitamin D utilization and chronically low reserves of this vitamin [41,42]. Taken together, these effects of vitamin D over-utilisation by combating glutaminergic and Ca++ excitotoxic damage and oxidative stress in retinal and visual pathways [43] very-likely set in motion a cycle of elevated arousal via the hypothalamic pituitary adrenal (HPA) pathway, resulting in still more noradrenaline release (with excessive noradrenaline release known to also disrupt sensory processing pathways [44]).

Thus in a chronic state, continued draw-down on the adrenal to produce even more catecholamines, may result in further overutilization of vitamins and eventually adrenal fatigue, with reduced neural transmission that further hinders visual and auditory sensory processing performance [45]. To summarise: primary nutritional-biochemical-oxidative stress imposes sensory processing strain, resulting in secondary HPA activation, resulting in mandated dopamine and catecholamine synthesis that then remotely draws down on vitamin D reserves. This demonstrates the potential for a vicious cycle of poor nutrition → low Vitamin D → low glutathione → increased oxidative stress and reduced GAD 67 expression, with low GABA and neuronal calcium neuro-excitotoxicity, followed by visual pathway damage → sensory strain → HPA axis activation → elevated catecholamines → further increased vitamin D utilisation → back to decreased vitamin D availability with low glutathione and high calcium excitotoxic neuronal damage explaining the underlying pathology of the hidden visual deficits in schizophrenia. Further research in this area is required [46].

### Rank II: Auditory domain

The auditory domain demonstrated the second strongest results on ROC analysis.

This domain contained biomarkers of reverse digit span (auditory (verbal) working memory), competing words (intra-cerebral dichotic listening), and delayed speed of auditory processing. This domain was also significantly correlated with the elevated catecholamine domain, implying that deficits in this domain also cause increased activation of the HPA, resulting in further elevated catecholamine release. This domain was the most strongly related to nutritional-biochemical abnormalities such as low folate, low vitamin D and high vitamin B12, with low vitamin B6 demonstrating a strong specific translational relationship.

The finding of low reverse digit span (impaired auditory working memory) has been previously documented for schizophrenia [47]. This project also utilised the (SCAN-3) test [48] as a measure of auditory processing disorder and an unsuspected finding was the prevalence of impaired competing word deficit (representing dichotic listening disorder) in the participants with schizophrenia and schizoaffective disorder. Subsequently, we found that dichotic listening deficits have also been previously reported in schizophrenia [49] and that reduced auditory speed of processing has been reported in chronic, non-paranoid schizophrenics [50]. Other literature indicates that reduced auditory processing speed is implicit to these disorders themselves and not attributable to anti-psychotic medication [51,52].

Low folate has been associated with sudden sensorineural hearing loss and folate supplementation assists hearing in older adults [53,54]. The damaging effects of Vitamin D lack on neural circuitry and sensory end organ function has already been described in the visual domain section above and this damage may well apply to auditory sense organs and neural circuitry as well. Low vitamin B6 was specifically related to dysfunction in the auditory domain, which is understandable, since this vitamin is a rate-limiting cofactor for tyrosine hydrolase and for the dopa-decarboxylase enzyme (aromatic L-amino acid decarboxylase) which converts L-DOPA into dopamine [55,56]. In a setting of delayed auditory function and HPA activation we theorise that there is an excessive need for the brain cells and adrenal gland to synthesise dopamine. Draw-down on vitamin B6 reserves for dopamine synthesis could be predicted to be high and since dopamine is a key activating neurotransmitter for the ear and auditory pathway neurotransmission, it is understandable that deficiency due to over-utilisation of this vitamin disturbs auditory activation and neural transmission [57].

### Rank III: Catecholamine domain

This domain demonstrated a strong correlate with the Symptom Intensity Rating (SIR) index and also produced a strong result on ROC analysis - findings consistent catecholamine elevation being integral to symptom-formation and with the monoamine hypothesis of schizophrenia [58]. However, in contrast to the dopaminergic hypothesis of schizophrenia [59], within the catecholamine domain, elevated noradrenaline levels predominated over elevated adrenaline levels and levels of both generally predominated over dopamine levels. Both dopamine and noradrenaline have been found to be raised in the blood of medicated and non-medicated patients with schizophrenia [60] and elevated noradrenaline levels have been reported in cerebral spinal fluid of both drug-free and neuroleptic-medicated schizophrenic patients [61]. Sustained, elevated levels of noradrenaline and adrenaline have also been reported in post-traumatic stress disorder [62], which is thought to be a traumatic response to inner confusion and anxiety caused by thought disorganisation and hallucinatory experiences [63]. We therefore theorise that this finding of peripherally elevated urinary catecholamines may not only cause sensory processing strain, but may also be reflective of dual visual and auditory sensory strain becoming a subliminal dynamic that is also an important component of secondary HPA activation, leading to further sensory signal disruption and psychosis-formation in schizophrenia and schizoaffective disorder. This interpretation partly fits with the 1990 findings of Adler LE, et al. [64] and also with the finding of stress-related pituitary enlargement in treated and untreated patients with schizophrenia [65-67]. It raises further important issues in the relationship to evolutionary and developmental origins of schizophrenia [68]. Raised catecholamines are also reported to suppress immune system responses [69,70], a finding that fits with reported reduced microglial activation in schizophrenia [71]. Noradrenalin and adrenaline elevation appear associated with mixed behavioural symptoms of anxiety and vigilant aggression that co-occur together with negative symptoms [72] and it is understandable that draw-down on both vitamin D and B6 (and even on folate reserves), may occur under the HPA axis mandate to synthesise more catecholamines as a secondary response to primary auditory or visual sensory strain. Evidence also exists that whilst low levels of Noradrenaline positively modulate cortical neurotransmission, at greatly increased levels, Noradrenaline neurotransmission is disrupted [72-74] which can be considered as a basis for fronto-temporal dysconnectivity, attentional disorganisation with impaired frontal cognitive function [75,76], temporal isolation and psychosis.

High Cu/Zn ratio and low vitamin D levels within the overall nutritional-biochemical domain exert strong translational effects upon the elevated catecholamine domain. This is to be expected, since copper is a cofactor for noradrenaline synthesis from dopamine and reports exist of high copper and low zinc associated with noradrenaline excess in schizophrenia [77,78]. Animal models also demonstrate vitamin D deficiency in relationship to elevated brain dopamine and noradrenaline levels [42]. The means by which vitamin D reserves may be over-utilised through combating the effects of catecholamine-inducted parathyroid hormone activation, has already been described [40,41].

In this study low vitamin B6 was specifically related to high dopamine levels, which is understandable since B6 is required as a cofactor at two enzyme levels as a cofactor for dopamine synthesis by tyrosine hydroxylase and for conversion of L DOPA into dopamine by the enzyme DOPA decarboxylase. The latter reaction is extremely important since L-DOPA is the only peripherally synthesized catecholamine capable of entering the blood brain barrier so its synthesis is the rate-limiting step for noradrenalin synthesis within the brain [55,56]. Apart from low vitamin B6 from poor nutritional intake in poorly functioning schizophrenic states [79], low vitamin B6 findings may be due to increased utilization of B6 under HPA imperative to increase L-Dopa and dopamine synthesis, triggered by sensory-strain-HPA-system-activation in order to manufacture more noradrenaline [80]. Given such an HPA imperative to synthesise more noradrenaline, the numerous research findings of elevated dopamine in schizophrenia, leading to the dopamine hypothesis of this condition [59] may just be reflective of dopamine’s role as a necessary precursor of noradrenaline.

### Rank IV: HPL/creatinine domain

Hydroxyhemopyrroline-2-one (HPL) emerged as a legitimate biomarker on ROC analysis. In a similar manner to translational relationships for catecholamines, HPL received strong translational influence from high Cu/Zn ratio and low vitamin D levels. Pyrrole products are ubiquitous in tissue biochemistry and HPL’s presence in urine is linked to oxidative stress and to low levels of vitamin B6 and zinc [81], which can further deplete B6 reserves and elevate free copper levels, respectively. Urinary HPL is thought to arise as an abnormal product of haeme metabolism induced by haemeoxidase under conditions of extreme oxidative stress where the porphyrin moiety of haemoglobin becomes degraded or where there is abnormal porphyrin synthesis [82-85]. When injected into rats and mice, HPL causes reduced activity, head twitching and backward locomotion (retreat) behaviour [86] and patients with elevated HPL levels report symptoms of anxiety, depression, low stress-tolerance and mood swings. HPL may also be stress and catecholamine-related and as such, elevated levels may be disorder-non-specific [87]. The structure of the HPL molecule has belatedly been discovered [88] and these findings invite further investigation of this oxidative, haeme-related molecule and its reported psychiatric effects.

### Rank V: Nutritional-biochemical domain

biochemical/biochemical biomarkers such as elevated free copper: zinc ratio, low vitamin B6 activation, low vitamin D and low folate levels, which on the “divide by three” test failed to exert sufficient individual strength to gain firm diagnostic association, were still able to demonstrate sufficient cumulative strength in terms of odds ratio of diagnostic association to have significant correlation with symptom severity.

While red cell zinc failed to produce an independent finding on ROC analysis, elevated serum free copper, in ratio with red cell zinc, was a biomarker of schizophrenia or schizo-affective disorder, confirming the hair-analysis findings of Ghanem et al. in 2009 [77]. Elevated free copper contributes to neuronal damage in circuits responsible for sensory-processing [89] and copper levels may also be influenced by reduced ceruloplasmin (copper binding protein) levels which has been linked to psychosis [90]. Free copper is redox active in the body and also contributes to reactive oxygen species and oxidative stress [91]. It also blocks the action of cystathione beta synthase (CBS) (Figure 1), an enzyme located upstream of glutathione production, preventing the formation of this major antioxidant for the brain [92]. Copper also binds to cholesterol producing an oxidative product 17-hydroxy cholesterol that is toxic to neurons [93]. Given these multiple neurotoxic and oxidative stress-related effects of elevated free copper, it is understandable that it can play a strong role in degradation of neurones in sensory-processing circuitry.

Zinc plays a reciprocal role to copper since it competes for metallothionein binding protein and acts as a defence against copper by inhibiting its absorption from the gastrointestinal tract [94]. Copper is a cofactor for the enzyme which synthesises noradrenaline from dopamine (Figure 1), which explains why low zinc facilitates high free copper which is then associated with elevated norepinephrine [78]. Animal models also suggest that zinc facilitates copper/zinc superoxide dismutase in blocking cell apoptosis signalling [95] and that zinc deficiency impairs fatty acid and myelin composition [96,97].

This research found that schizophrenic and schizo-affective patients may be extremely sensitive to inadequate levels of activated B6, since its ROC determined cut-off value occurred within the normal laboratory reference range. This is theoretically due to the many co-factor roles of B6 across biochemical pathways related to monoamine synthesis and prevention of oxidative stress. Indeed, inadequate levels of activated B6 may be reflective of its increased utilisation in these multiple biochemistry pathways. For instance, in the initial stages of dopamine synthesis, the enzyme tyrosine hydroxylase requires B6 as a cofactor [98] and downstream to this, L-Dopa is the only catecholamine precursor to enter the central nervous system being converted into dopamine by the enzyme DOPA decarboxylase, with vitamin B_6_ as a required cofactor for this reaction (Figure 1), [55,56]. In the transulfuration pathway (Figure 1) the enzymes cystathione beta synthase (CBS) and cystathionase also require vitamin B6 for the synthesis of the brain antioxidant glutathione via [99]. Such B6 over-utilization with relative deficiency may then inhibit the action of the enzymes CBS and S-adenosylhomocysteine hydrolase (SAHH) [100], leading to back-ups in the transmethylation cycle and methylation cycle with elevation of homocysteine and S-adenosyl homocysteine (SAH) [101], respectively. Indeed, when such effects coincide with free copper toxicity and further CBS inhibition [89,91], glutathione synthesis may then be drastically reduced and free radicals damage neuronal circuitry made vulnerable in this setting of oxidative stress. In such a setting, high copper also promotes further noradrenaline synthesis from dopamine [78] whilst elevated SAH contributes to raised catecholamine levels by directly inhibiting cathechol-o-methyl-transferase (COMT), [102-104]. With inhibition of this COMT enzyme that is responsible for the final step of catecholamine metabolism (Figure 1), catecholamine intermediate metabolic products accumulate and break-away metabolism of backed-up intermediate-metabolites result in abnormal neurotoxic products and further cytotoxic neuronal consequences [105].

In this study methyltetrahydrofolate reductase (MTHFR 677 T) polymorphism demonstrated a positive significant correlate for high homocysteine levels, however MTHFR polymorphism, along with plasma homocysteine, urine HVA and MHMA were not successful as biomarkers on ROC analysis. Though weak enzyme dynamics due to thermo-lability of the MTHFR 677 T polymorphism has received much research attention with regard to schizophrenia and homocysteine elevation [106], there is evidence that contradicts this finding [107]. The MTHFR polymorphism is linked to deficiency of its product 5-methyltetrahydrofolate (5-MTHF) and in settings where enzymes in other homocysteine metabolism pathways are also inhibited, backed-up, elevated homocysteine may occur (Figure 1). Such findings have been reported in schizophrenia [106] and it may be that our ROC finding of elevated vitamin B12 represents a compensatory reaction of unknown mechanism designed to assist methionine reconstitution from homocysteine. In a setting of MTHFR polymorphism homozygosity with lack of 5-MTHF product, elevated vitamin B12 may be a necessary compensation in order to sustain methylation cycle precursors for maintenance of S–adenosyl methionine (SAMe) levels (Figure 1).

Low red cell folate was also a biomarker in this study and this finding has been previously reported in schizophrenia [108] where poor diet may convey vulnerability for low methylation and low SAMe (Figure 1), [109]. SAMe also stabilises CBS in the transulfuration pathway, protecting this pathway against glutathione depletion and therefore against oxidative stress [110].

Folate and vitamin B12 (cobalamin and methylcobalamin) have important roles in sustaining production of SAMe, which in turn serves to prevent oxidative stress and catecholamine elevation by serving to stabilise CBS and also as a cofactor for catechol-o-methyltransferase’s (COMT) metabolism of catecholamines [111]. Therefore relative unavailability of SAMe due to colluding influences of homozygous MTHFR polymorphism, low folate or low B12 levels may allow oxidative stress and impair COMT activity, explaining our correlate and ROC findings of elevated HPL and elevated catecholamine levels. The effects of oxidative stress and elevated catecholamine levels have been well documented in literature and are also noted in psychotic children [112-114].

In contrast to the above findings and their biochemical interpretations, other research findings have not found notably elevated homocysteine levels in schizophrenia [115]. Indeed in this research project, homocysteine failed to reach ROC biomarker status and this may be noteworthy in the collateral setting of positive ROC’s findings for elevated free copper to zinc ratio. This is because elevated free copper inhibits the homocysteine metabolising enzyme (cystathione-beta-synthetase (CBS) in the transulfuration pathway leading to glutathione synthesis [91]. A further plausible explanation is that since vitamin B6 is required for homocysteine synthesis, low vitamin B6 may accompany low homocysteine. If this is so, then the causal explanation that COMT inhibition in schizophrenia may occur via SAMe unavailability for COMT facilitation [111] is less likely and an alternative explanation is required. Since SAH-hydrolase (SAHH) requires vitamin B6 for S adenosyl homocysteine (SAH) conversion to homocysteine (Figure 1), SAH is more likely to accumulate in a setting of low vitamin B6 [101]. Then in a setting of retrograde, elevated SAH, the remotely situated COMT enzyme which metabolises catecholamines is directly inhibited [103,104]. Taken together, these explanations bring together our ROC biomarker findings of elevated catecholamines, low vitamin B6, high free copper and high vitamin B12, providing a more cohesive dynamic understanding of potential interactions and causes. Finally, the accompanying finding of failed biomarkers for urine HVA and MHMA reflects the tendency for elevated catecholamine intermediate metabolic products to find break-away metabolic pathways in a setting of their inhibited end-stage COMT metabolism. These abnormal metabolic pathways unhappily resulting in neurotoxic intermediate metabolites which can contribute to loss of neuronal density in schizophrenia [105,116].

In summary, visual and auditory sensory processing systems in this study demonstrate peripheral deficits and prematurely aged, delayed processing that appears translationally related to folate, B6 and D vitamin-deficiency, copper-related neural excitotoxicity and oxidative stress. These abnormalities then accumulate to contribute to the fundamental architecture of schizophrenia and schizoaffective disorder. Sensory processing strain may then initiate HPA axis activation, leading to a cycle of elevated catecholamine synthesis with inherent overutilization of vitamin cofactors and further depletion of their body reserves. This then initates a runaway process of catecholamine elevation, which may occur in a setting of S adenosyl homocysteine (SAH) inhibition of the catechol-o-methlyltransferase (COMT) enzyme. Inhibition of this catecholamine-metabolising enzyme then allows to build-up of elevated catecholamines and break-away neurotoxic catecholamine metabolites that inflict further damage on sensory neurones (Figure 3).

### Limitations

One focus of this research was to use biomarker discovery to characterise schizophrenia and schizoaffective disorder. To avoid confounding variables many exclusion criteria were imposed which slowed recruitment and resulted in a small discovery data-set. Small sample size, together with non-normal data distribution for many variables, precluded use of principal component analysis, latent variable analysis and multivariate analysis. ROC analysis and Spearman’s analysis were used instead to identify promising biomarkers and their relationships. Sequentially collapsing the data through ROC analysis and then regression analysis, has potential for loss of some data strength. Though case–control study designs have inherent susceptibility to prevalence and selection bias [117,118], they are allowable for a discovery data-set [119] and have a respectable record in discovery research [120] for low period prevalence disorders, such as schizophrenia (0.35 per 1000) [121].

Difference in selection processes may well have been offset by the random nature of success in the recruitment process itself, incurred by participant failure to meet the multiple exclusion criteria combined with a refusal-to-consent ratio of 4 to1, for approached patients and controls alike. Also, the application of this design to only one bracket of psychotic disorders (schizophrenia and schizoaffective disorder) in this project is limiting, since it is not known whether the ROC variables discovered for these conditions are disease specific or also occur in other mental illness states.

The use of the DSM IV as a diagnostic tool for case selection may also be a confounding variable, since diagnoses may range across spectrum and boundaries and thresholds for diagnosis are yet to reach optimal standards for categorisation [122].

A further limitation of this project was that assessors undertaking rating scales and neurophysiological measures, were functioning in a real-world clinic-setting, so were not blind to participants’ patient or control status.

The full effect of confounding variables upon results of this research was also not fully assessed. For instance, although rigorous efforts were made to exclude substance-related diagnoses, it is known that alcohol misuse is often under-reported by patients [123], and has a known association with low folate, high B12 and elevated catecholamine levels [124,125]. It was not possible to control for participant smoking in this project and smoking also has been variably reported to effect monoamine oxidase levels [126], which can theoretically influence monoamine levels. Though fasting biological samples were collected for this study, there was no longer-term control or assessment of dietary intake, which, in principle, could affect reserves of vitamins, minerals and monoamines [127]. Though participant assessments took place within a 4 day window, it is possible that variance in relationship to a patient’s stress or activity state, may have effected monoamine levels and other related-variables, over the period of assessment [128].

Despite biochemical pathway linkages and the strong correlation of elevated catecholamine biomarkers with Symptom Intensity Rating (SIR) as an indicator of clinical severity, the role of medication as a confounding variable in this project cannot be fully excluded. Although there is widespread research evidence that antipsychotic medications increase catecholamine metabolic turnover and reduce dopamine and noradrenaline levels [129], evidence regarding antipsychotics such as Quetiapine is less certain [130]. By blockage of the dopamine-2 receptors and antagonism of alpha 1 adrenergic receptors Quetiapine contributes to suppression of hyperdopaminergic-related positive symptoms in acute schizophrenia [131], 132 and brings about profound hypotension in overdose, from which reversal and rescue can only be achieved by noradrenaline [132]. However this antipsychotic has been contrastingly reported to increase dopamine and noradrenaline levels in the rat cortex [133]. The exact extent of pharmacological action of sodium valproate is also inconclusive. From its action on GABA, and its efficacy in mania treatment, the mechanism of sodium valproate action is presumed to involve reduction of catecholamine levels. Also, since catecholamines normally promote beta oxidation of fatty acids, reduction of catecholamine levels by valproate is proposed as the mechanism for valproate-associated weight gain [134]. However, evidence from rat and ox brain experiments suggests that sodium valproate may increase monoamine levels [135,136] whilst a contrasting trend is reported from mice experiments [133] and a human CSF study indicates that sodium valproate may increase dopamine metabolism [137], theoretically decreasing dopamine levels.

Although the ubiquitous, oxidative-stress related molecule HPL earned its place as a discovery biomarker additional biomarkers for oxidative stress, future candidate markers could be widened to include other markers of oxidative stress. Similarly, other neurotransmitters and measurement of SAMe:SAH levels and COMT activity levels that would further validate putative biochemical linkages.

Elevated urinary catecholamine biomarkers identified in this study cannot be directly related to brain neurotransmitter changes, since fully-synthesized monoamines do not cross the blood–brain barrier even though their precursor substance, L-Dopa is capable of this transition [80]. Spot-urine collection, as a method for peripheral monoamine analysis, has also attracted criticism [138] though it is practical in a psychiatric setting, is successful in paediatric settings [139,140] and is gaining ground as a useful analytic method relating to body biochemistry [141].

Due to all these considerations, the optimal means for replicating and validating this project’s discovery biomarkers is via a large, well-characterized, prospective, multi-site-clinic trial on a single series of consecutive patients, with blinded assessors and an emphasis on collecting data from ultra-high risk medication naïve subjects.

## Conclusions

This research is a pioneering effort to quantify biomarkers for schizophrenia and schizoaffective disorder across several domains of function. It introduces an objective, quick, simple-to-apply clinical assessment tool for understanding a patients underlying pathology. Biomarkers were discovered for some variables such as dichotic listening disorder and asymmetric distance vision that are not easily clinically recognised and therefore represent largely unmet needs within current clinical assessment and practice for psychosis. Since remediation techniques are already in existence for these disorders, such findings hold potential for reduced treatment-resistance, cost-burden, social stigma and improved relapse-prevention.

Correlative findings demonstrate that schizophrenia and schizoaffective disorders relate to a matrix of subtle, sub-clinical disorders ranging from the intra-cellular nutritional biochemistry dysfunction to oxidative stress and elevated catecholamine neurotransmitter levels. These then link up translationally with dysfunction in both peripheral and central domains of neuro-sensory processing.

Visual processing tracts are damaged and their processing is delayed in schizophrenia and schizoaffective disorder. This has a strong bidirectional relationship to catecholamine excess, particularly of noradrenaline and a moderate relationship to pathology in cellular nutritional biochemistry and oxidative stress domains.

Auditory processing tracts are also substantially delayed and dichotic hearing is adversely affected. Moreover, this damage is most strongly related to pathology in the nutritional domain of biochemistry.

The implications from this research is that visual and auditory sensory processing systems are prematurely damaged and aged by vitamin-deficiency, copper-related neural excitotoxicity and oxidative stress and that deficits in these abnormalities fundamentally contribute to the biochemical architecture of schizophrenia and schizoaffective disorder. Sensory neuronal damage and strain may then have secondary potential to initiate HPA axis activation, followed by a cycle of elevated catecholamine synthesis with inherent overutilization of vitamin cofactors and depletion of their body reserves. This initates a runaway process of catecholamine elevation where break-away neurotoxic metabolites, high free copper and oxidative stress combine to further ramp up dysfunction of neurosensory pathways. Subliminal recognition of processing strain within these pathways then activates the hypothalamic pituitary adrenal axis, which together with high free copper levels, pushes catecholamine synthesis towards higher and higher noradrenaline levels. Eventually ramped-up levels of a noradrenaline induce disconnection in visual and auditory sensory pathways culminating in sensory attentional disorganisation, low visual span and frontal and temporal isolation with psychosis.

Although current research indicates that many of these findings occur irrespective of medication, validation of these biomarkers is warranted in ultra-high risk medication naïve subjects in a larger, well-characterized, cohort with blinded investigation. Deeper understanding of biomarkers will widen and objectify our understanding of psychosis and improve targeted remediation of the substructure of schizophrenia and schizoaffective disorders.

## Methods

### Study design

A case–control study design and multiple exclusion criteria, were used to select cases and exclude organic causes and variables which could influence outcome measures for selected markers. In this manner, we reduced possible aetiologies of schizophrenia and schizo-affective disorder to their core functional conditions. Using such highly characterised cases, we hoped to unmask some of the multiple, subtle, cumulative variables that could contribute to these disorders.

### Participants

All participants were informed of the goals, assessment procedures and funding of this study and provided written consent. Ethics permission for the study was obtained from the hospital ethics committee. Participants were recruited between 2010 and 2014 from The Queen Elizabeth Hospital, Woodville, South Australia and from satellite mental health clinics in the western catchment area of Adelaide, South Australia. They were from multi-ethnic backgrounds with an age-range between 18 and 60 years of age. Similarity of psychotic symptoms occurs in both schizophrenia and schizo-affective conditions and their diagnoses were made with roughly equal incidence in the project setting at a 1.2 to1 ratio, respectively. Recruitment of both psychotic conditions allowed for sufficient recruitment numbers to occur under the strict exclusion criteria imposed, whilst also allowing scope for later analysis of depressed or manic affective symptoms in relationship to biomarkers discovered. Recruitment and assessments were conducted directly and by phone across hospital, research institute and community clinic settings. Control participants were recruited by phone from a volunteer population associated with the hospital and drawn from the same catchment area as patients.

### Patient recruitment and sample selection

Non-detained ward patients in partial remission but with residual symptoms of psychosis, were recruited and assessed in the expected last week of their admission, by which time they were in a less severe state of psychosis and sufficiently recovered to give informed consent. After 7 early dropouts due to declining mental state, a total of 82 symptomatic participants (cases) were recruited and completed assessment. Early statistical analysis of confounders required that 15 participants on SSRI or SNRI antidepressant medication were excluded from further analysis, due to their suppressive effect on catecholamine levels, therefore the number of participants used in the final statistical analysis was 67.

Patients were included in the study if they received a diagnosis of schizophrenia or schizoaffective disorder, made by a consultant psychiatrist in the ward or community satellite clinic setting, DSM IV-R diagnoses were checked against a DSM-IV-R symptom checklist at the recruitment stage, in order to confirm that a correct diagnosis had been made. Clinical appraisal of a patient’s judgment capacity and orientation in time, place and person was undertaken during recruitment, in order to confirm capacity to consent and exclude delirium. Informal examination to exclude ocular-muscle dysfunction, hand, forearm and shoulder dysfunction was also conducted at this stage, in order to ensure that a patient was free to proceed to neuro-physiological tests in the absence of rigidity, dyskinesia, tremor or postural instability as a result of extra-pyramidal side-effects from antipsychotic medication [142]. Persons medicated with Clozapine, Olanzapine, anti-histamines or vitamin therapy were excluded, since these variables had potential to confound biochemical results for one of the candidate markers. Participants taking antipsychotic-agents with lesser-effect on histamine receptors (Zuclopenthixol, Modecate, Amisulpride (Solian) and Risperidone) were however included. The only exception to this was the inclusion of seven patients on Quetiapine. Mood stabilisers were allowed and four patients received sodium valproate. Pharmacotherapy of patients remained stable during the assessment period which was usually completed on the same day as recruitment and in a few cases, within four days of recruitment. Patients were also excluded from the study if they had a recent or unresolved history of alcohol or other substance abuse, since this can confound biochemical and particularly neurotransmitter results. Persons with upper respiratory tract infections, visual or auditory disability were excluded since these conditions could effect auditory or visual sensory processing tests. Persons with intellectual, clinically-documented or descriptive-history of head injury with unconsciousness or hospitalisation, were also excluded, since these conditions may effect ability to comply with tests or be complicated by psychosis. It was considered impossible to control for smoking as a variable and have any chance of sufficient patient recruitment.

In order to minimise severity bias by comparison of very-sick with well controls in this case–control study, ward patients were recruited within a week of their expected discharge back into the community from which controls were drawn and selection bias was minimised by recruiting some clinic patients by phone and assessing them in the same community setting as the recruited controls.

### Control recruitment and sample selection

A total of 72 control participants, were recruited with the assistance of the Population Research and Outcomes Studies (PROS) Unit of the University of Adelaide. These participants were volunteers, from the same catchment area as patients, affiliated with the Department of Medicine and the North West Adelaide Health Study (NWAHS), for research purposes. Using the same exclusion protocol as for patients, these participants were age-stratified and randomly recruited by phone contact, over the same assessment period as patients. In a low prevalence condition such as schizophrenia with a period prevalence 0.35 per 1000,none of the controls reached threshold for schizophrenia or any DSM diagnosable mental illness, but they were rated for any subclinical symptoms by a psychiatrically-trained assessor who was not blind to their asymptomatic status, but was blind to all biological test results at the time of rating. Five presenting control participants were assessed, but had their data excluded due to their not meeting basic vision or hearing criteria for participation in the study. In order to obtain younger controls for age and sex matching, two asymptomatic volunteers who resided within the same NWAHS catchment area, were recruited from a local surf life-saving club. Although recruitment methods differed between cases and controls, the control sample used for the final analysis was drawn from the same catchment area and outpatient environment as the patients and the sample for final analysis consisted of 67 cases and 67 (72–5) controls for which baseline sample characteristics are provided in Table 1.

### Other participant assessment procedures

Together with a review of the participant’s case notes, a standard interview protocol collected demographic information and information related to development, organic, biochemistry and sensory-processing disorder. Also recorded was absence or presence of developmental difficulty or learning delay, medical co-morbidity, and head injury, family history of mental illness, glasses or hearing aids. Symptoms in the Brief Psychiatric Rating Scale (BPRS) and the Positive and Negative Syndrome Scale for Schizophrenia (PANSS) - were amalgamated in the interest of reducing assessment time. Using this rating tool, each symptom was rated from 1 to 7 for intensity. These ratings were then summated to give an overall symptom-intensity-rating (SIR) index, which was taken as a measure of clinical severity. Hospital and clinic ratings were made by psychiatrically trained registrars, who were blind to index laboratory and sensory-processing test results, but not to patient status, at the time of rating. Ratings were checked by DSM diagnostic checklist. Rating for control participants were made by one psychiatrically-trained assessor. It was not considered practical in the real world, for raters to be blinded to the diagnostic status of the participant, since many patients were unable to mask their condition, due to residual psychotic symptoms.

### Sensory processing assessment

Reverse digit span working memory and spatial working memory were selected because of their known association with schizophrenia [143,144]. Other sensory processing tests were selected based upon clinical experience with patients with schizophrenia, where visual and auditory processing speed deficits had been observed in many cases and suggested that cross-talk between auditory processing disorder and schizophrenia might occur.

**Figure 4:**
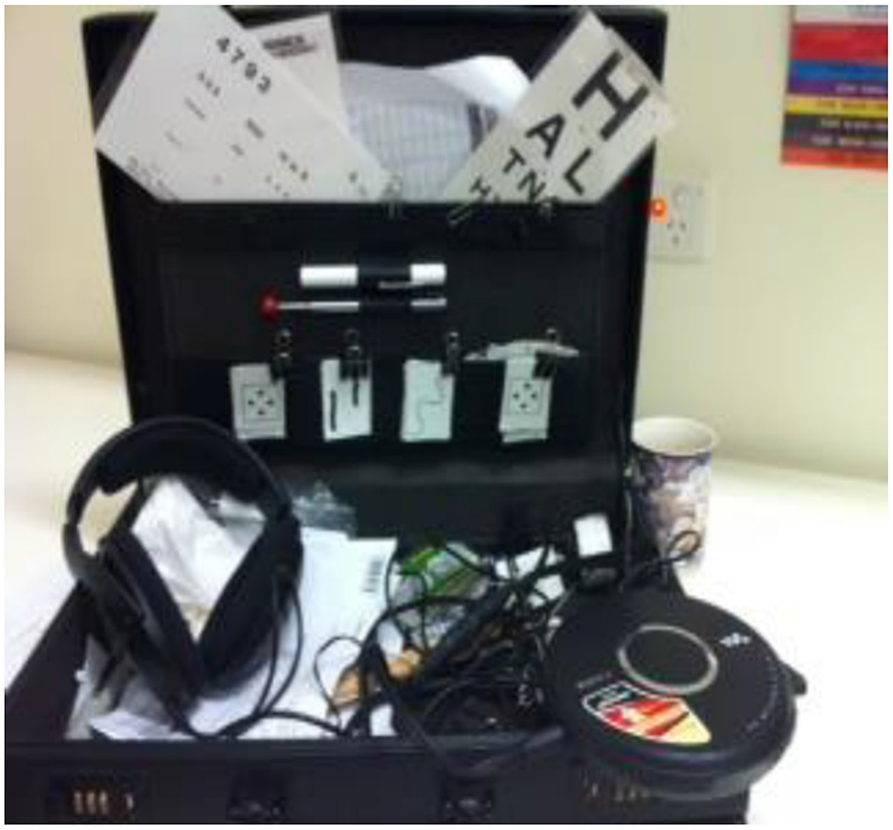
**Office assessment equipment.**

**Figure 5:**
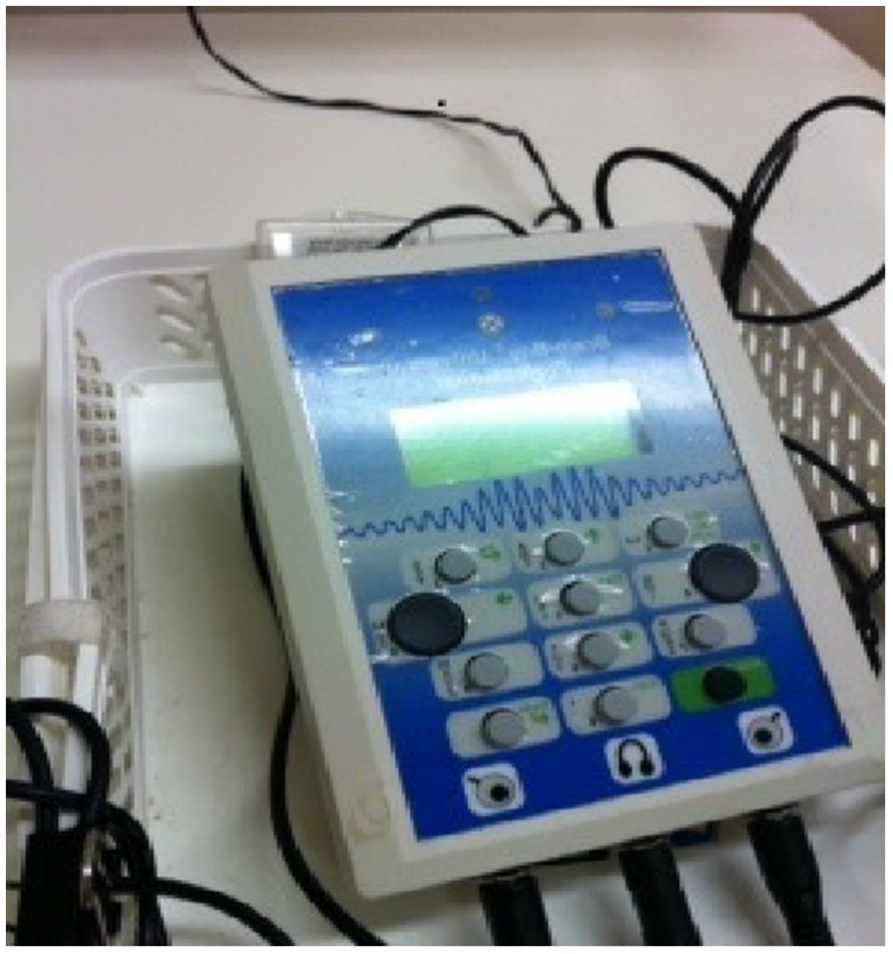
**Sensory speed of processing equipment.**

After determining visual and hearing acuity, all participants were assessed for selected sensory and cognitive variables related to auditory and visual processing. Assessments were conducted in auditory and visual domains, at a time separated from both blood and urine collection and within two hours of such biological sample-collection. The methods of sensory processing assessments for this project are outlined in Tables 5 and 6 and shown in Figures 4 and 5.

**Table 5:** **Summary of visual assessment methods**

**Assay/Assessment**	**Method**	**Laboratory/Reference**
**Visual**		
Near vision acuity test	Sussex Vision test of near vision. Near vision test card SNT-3000-L, 2009–2011.	Sussex Vision International Ltd. (35)http://sussexvision.co.uk/index.php/near-tests/reading-tests.html.
Visual (symbol) span	Increasing number of symbols are presented in a standardised order from left to right. Test score reported as the absolute number of visual symbols recalled in the correct order.	Based on Visual Symbol subset test of WMS-IV (Weschler 2009)
Distance vision **(**Binocular distance vision acuity)	Right distance vision, then left distance vision, with 20 seconds inter-test interval.	The Snellen-Chart (Snellen 1860)
Threshold visual speed of processing performance as a percentage of age	Person tested sees two brief flashes of light randomly presented from left-to-right or right-to-left on multiple occasions, and must decide which light flash appeared first. The inter-stimulus time interval (ISI) between the flashes is shortened by computer algorithm, if the answer is correct, otherwise it is lengthened. A performance-age rating, is provided, configured against norms-for-age. Performance-age is subtracted from the individual’s actual age and the result divided by the age of the test subject is multiplied by 100.	Brain Boy Universal Professional instrument (MediTECH 2010)
(Expresses visual processing speed in terms of the visual processing system’s relative age)		
Shortest interval of time a person can notice between the order of presentation of two optical stimuli. Speed of visual order processing increases with age. For adults between the range of 18 and 60 years, the normal range for visual speed of processing is 24 to 72 milliseconds). For adults between the range of 18 and 60 years, the normal range for visual speed of processing is 24 to 72 milliseconds.

**Table 6:** **Summary of auditory assessment methods**

**Assay/Assessment**	**Method**	**Laboratory/Reference**
**Auditory**		
Reverse digit span	With gaze aversion by listening participant and tester, digits are read in set sequence. The tested participant is asked to repeat them in reverse order. Reported as the absolute number of digits correctly recalled in reverse order.	Subset of Wechsler Adult Intelligence Scale III (Wechsler 1997)
(Measures auditory (verbal) working memory)		
Normal range is 6 to 7		
Competing words performance for age as a percentage of age	A voice-over CD and earphones test ability to correctly identify both of two competing-words (CW), delivered separately to the right and left ears. Using this test’s normative-for-age database, the difference between each test subject’s expected and actual performance-for-age was calculated, and this was then divided by the actual age of the test subject, and multiplied by 100.	SCAN-3 (Keith 2009)
(Intra-cerebral dichotic listening performance for processing of auditory information)		
Normal ranges vary with age		
Threshold speed of Auditory processing as a percentage of age	Person tested hears two clicks, randomly presented from right to left and left to right side, presented through headphones. By pressing a right or left button, a decision must be made from which side the dual-stimulus originates. If the answer is correct, the inter-stimulus interval between flashes (ISI) is shortened, otherwise it is lengthened. The auditory order threshold is the shortest ISI a person can correctly differentiate between two auditory impressions. A read-out of the threshold speed of auditory (order) processing is provided, along with a norm performance-age rating. Auditory speed of (order) processing performance as a percentage of age is calculated by subtracting the norm-for-age from the performance-age, divided by the age of the test subject, multiplied by 100.	Brain Boy Universal Professional instrument (MediTECH 2010)
(Speed of auditory processing systems relative to age)		
Speed of auditory processing reduces with age. For adults in the age range of 18 and 60 years, the normal range for auditory speed of processing is 46 to 72 milliseconds.

Where applicable, visual assessment was conducted using the participant’s usual glasses and alternate-cover-test was conducted prior to visual testing, to exclude visual fixation disparity (phoria or tropia) as a potentially confounding variable [145]. Visual assessments included near and distance visual acuity, visual attention span, speed and accuracy of visual processing.

Auditory assessments (Table 6) were conducted in a quiet room (ambient noise level 20 dB) and preceded by examination of the external auditory meatus to exclude obvious pathology or sebum obstruction. Audiometry examination was conducted using the MAICO Audiogram MA 40 [146], at 250 Hz to 4000 Hz to determine hearing deficits (defined as air-bone conduction gaps > 10 Hz and/or threshold abnormalities > 500 - 1000 Hz) and laterality differences. Auditory processing assessment outcome measures were of acuity, attention, and threshold speed and accuracy of auditory processing. All assessments were performed by a neuro-psychiatrically trained assessor who was blind to laboratory results. It was considered impractical to blind the assessor to participant status, as residual symptoms of psychosis were obvious to the trained observer. Apart from the prerequisite audiogram assessment, equipment is compact and carried in a suitcase (Figures 4 and 5).

### Biochemical assays and specimen collection

It was considered important to use clinically-accessible, commercially-available tests to assay biochemical markers in this study. Investigation involved blood and urine samples collected from all participants. Biochemical collection methods are documented in Table 7. All biochemical-testing was conducted by independent commercial laboratories that were blind to participants’ case or control status and all participant raters and assessors were blind to laboratory results. The theoretical background for selection of biological markers are outlined below and elaborated in Figure 1.

**Table 7:** **Summary of biochemistry tests and methods with website links and literature citations**

**Test**	**Method, analyser, reagents**	**Laboratory/Reference**
**Neurotransmitters**		
Biogenic amines: dopamine, Noradrenaline and Adrenaline,	Spot-baseline (fasting) urinary neurotransmitter testing (second void morning), snap-frozen to minus 30 degrees and analysed by mass spectrometry, using nanomols per millimol of urinary creatinine as a standard.	SA Pathology, Adelaide, South Australia.
		Whiting MJ. 2009. Simultaneous measurement of urine metanephrines and catecholamines by liquid chromatography with tandem mass spectrometric detection. *Annals of Clinical Biochemistry*, **46**:129-136
Creatinine	Spot urine specimen from the same void as biogenic amines, expressed in (millimols per Litre)	SA Pathology, Adelaide SA.
**Oxidative stress: Urinary hydroxyhemopyrroline-2-one**		
Urinary hydroxyhemopyrroline-2-one (HPL)	Spot urine (second void morning) in ascorbic acid, snap frozen (−30C) and light-protected. Colorimetric method at 540 nm, following solvent extraction and reaction with Erich’s reagent. (micrograms per decilitre).	Applied Analytical Laboratories, 8/26 Nestor Drive, Meadowbrook, Queensland, Australia. +61 7 3133 1615.
**Nutrition-Biochemistry**		
Vitamin D ( 25-OH)	Diasorin Liason assay kit, for use on the Liaison platform (nmol/L).	Clinpath Laboratories, 19 Fullarton Rd, Kent Town. South Australia 5067 +61 8 8366 2000.
Serum total Vitamin B12	Competitive Electrochemiluminescent Immunoassay. Roche Modular E 170	Clinpath Laboratories. As above.
	Automated Immunoassay Analyser, using Roche Vitamin B12 Reagent (pmol/L).	
Red Cell Folate	Competitive Electrochemiluminescent Protein Binding Assay, using Roche Modular E 170, using Roche Folate Red Blood Cell (RBC) Reagent and Roche Folate RBC Haemolysing Reagent. on Automated Immunoassay Analyser (nmol/L).	Clinpath Laboratories. As above.
Vitamin B6 (Pyridoxal-5’-phosphate coenzyme form)	Whole blood High Pressure Liquid Chromatography with fluorescent detection. Chromsystems Vitamin B6 in Whole Blood High pressure Liqid Chromatography Reagent Kit. Waters Alliance 2695 Separations Module. Waters 474 Fluorescence Detector (nmol/L).	Sullivan Nicolaides Pathology
		143 Whitmore St, Taringa. Queensland 4068. Australia. +61 7 337 8666
Serum Copper	Flame Atomic Absorption Spectrophotometry.	Douglass Hanly Moir Pathology
	Varian AA-240FS (umol/L).	14 Griffnock Avenue, Macquarie Park.
		New South Wales 2113. +61 2 9855 5222.
Red Cell Zinc	Inductively coupled plasma mass spectroscopy (ICP-MS), using 6% n-Butanol reagent and Agileny ICP-MS 7500ce analyser (umol/L).	Sullivan Nicolaides Pathology.
		As above.
Serum Ceruloplasmin	Immunoturbidimetric method, using 6 K91-30 Multignet Caeruloplamin Kit and Abbott Architect ci16000 analyser (g/L).	Douglass Hanly Moir Pathology.
		As above.
Percentage Free Copper/Red Cell Zinc	Percentage of free copper in the serum calculated by an equation based on the molecular and atomic weights of ceruloplasmin and copper (one ceruloplasmin molecule binds to six copper atoms). The ratio of the percentage free copper to red cell zinc was calculated as “percentage free copper”/“Red cell zinc umol/L”.	Calculated by authors
**Intermediate substrates and enzymes**		
MTHFR Ala222Val (C677T) methyl tetrahydrofolate reductase polymorphism	Real time PCR analysis	Douglass Hanly Moir Pathology.
	Roche Diagnostics Light-Cycler 480 kit. Using TecnoBiol reagents, Sigma probes and primers on Roche LC480 analyser.	
		As above.
Plasma homocysteine	Ice transported EDTA sample. Competitive Chemiluminescent Immunoassay, using Seimens Homocysteine reagent on Seimens Advia centaur Automated Immunoassay (umol/L).	SA Pathology. +61 8 8222 3000 As above.
Serum histamine	Beckman Coulter Radio Immunoassay, using Beckman Coulter R.I.A. Kit on Perkin Elmer Wizard 1470 Automated Gamma Counter (umol/L).	Sullivan and Nicolaides.
		As above.

Baseline, fasting, spot-urine for biochemical assays of dopamine, noradrenaline and adrenaline as well as their metabolites homovanillic acid (HVA) and methoxy-hydroxymandelic acid (MHMA) and the serotonin metabolite 5-hydroxyindoleacetic acid (5-HIAA), were collected. As part of this analysis, urinary creatinine was determined as a urine concentration standard and this standard was also used for urinary hydroxyhemopyrroline-2-one (HPL) - a metabolite reported in schizophrenia that is considered to be indicative of oxidative stress. Fasting blood was also collected for vitamins and mineral cofactors and intermediate substances related to folate and methionine (one-carbon) cycles which have theoretical potential to exert cumulative effects on neurotransmitter synthesis and metabolism (Figure 1). These allied pathways are effected by enzyme cofactors vitamin B6, B12, red cell folate], serum copper and red cell zinc], with other substances such as plasma homocysteine, serum ceruloplasmin, serum histamine], and serum methyltetrahydrofolate reductase (MTHFR 677 C- > T) gene polymorphism. Assays were also performed for vitamin D, which has a proven epidemiological link with schizophrenia In order to ensure that urine neurotransmitter levels were not unduly affected by hypothalamic-pituitary axis (HPA) activation there was a minimum of two hours separation between blood collection and urine collection.

### Data analysis

Lack of normal distribution in the ROC variables necessitated the use of EsyFit software [30] to determine forms of data distribution and other characteristics such as mean, median and mean absolute deviation. Other statistical analysis was conducted using XLSTAT [29] for descriptive statistics, Receiver Operating Curve (ROC) analysis and odds ratio analysis of association of ROC variables and domains with a diagnosis of schizophrenia or schizoaffective disorder, at a 95 per cent level of confidence. A ROC curve is a graphical summary of discriminatory accuracy between a population with a disease and a population without the disease. The ROC curve describes the relationship between the true positive fraction (sensitivity) and the false positive fraction (one minus specificity) for different positivity thresholds [41,42]. Criteria for candidate markers to approach biomarker-status are a ROC AUC lower boundary of 0.6 and an odds ratio of > 2, at 95 per cent level of confidence. We also included outcomes for variables falling slightly short of these criteria which nevertheless contributed to raising the AUC within any particular domain. The strength, direction and covariance-potential of key ROC-established-variables and domains, were explored in relationship to other outcome-variables, using Spearman’s Correlation. Due to abnormal distributions and low sample size, principal component analysis and latent variable analysis were not undertaken. Lowess logistic non-parametric. Lowess (non-parametric, locally-weighted regression analysis), (available in XLSTAT) [147] was then used to explore predicted, translational inter-domain relationships for discovered biomarkers (Figure 2), where ideal significance of predictive probability is ≥ 60 per cent, for at goodness of fit (R 2) of ≥ 0.6.

## Abbreviations

DSM-IV-R: Diagnostic and statistical manual of mental disorders [16]
ROC analysis: Receiver operating curve analysis
AUC: Area under the ROC curve
SIR: Symptom intensity rating, as an index of clinical severity
MTHFR 677 C- > T: Methyltetrahydrofolate reductase gene polymorphism
HVA: Homovanillic acid
MHMA: Methoxy-hydroxymandelic acid
5HIAA 5: Hydroxyindoleacetic acid
COMT: Cathechol-o-methyl-transferase
SAH. S: Adenosyly homocysteine
SAHH. S: Adenosyl homocysteine hydrolase
SAMe. S: Adenosyl methionine
CBS: Cystathione beta synthase
HPL: Urinary hydroxyhemopyrroline-2-one
HVA: Hypothalamic pituitary adrenal axis
SSRI: Serotonin reuptake inhibitor
SNRI: Serotonin noradrenaline reuptake inhibitor
GABA: Gamma amino butyric acid
